# Traditional Chinese medicine trade among RCEP countries: structural characteristics and determinants

**DOI:** 10.3389/fpubh.2024.1508839

**Published:** 2025-01-07

**Authors:** Yue Fang, Meng Xue Tang, Xu Liu

**Affiliations:** ^1^School of Economics and Management, Anhui University of Chinese Medicine, Hefei, China; ^2^Anhui Institute of Conservation and Development of Traditional Chinese Medicine Resources, Hefei, China

**Keywords:** traditional Chinese medicine trade, regional comprehensive economic partnership, structural characteristics, influencing factors, social network analysis, quadratic assignment procedure

## Abstract

**Background:**

With the increasing global focus on health and the growing popularity of natural therapies, Traditional Chinese Medicine (TCM) products, including extracts, crude drugs, and herbal preparations, are widely utilized as both primary and complementary medicines worldwide. The Regional Comprehensive Economic Partnership (RCEP), spanning 15 countries across East Asia, Southeast Asia, and Oceania, offers a vast market for TCM. However, limited research has been conducted on the complex trade relations among RCEP members.

**Methods:**

The structural features and nodes attributes are analyzed using Social Network Analysis (SNA). Influencing factors are studied through the Quadratic Assignment Procedure (QAP) model. We also compiled a list of medicinal plants among RCEP member countries and the main TCM export products.

**Results:**

The scale of TCM trade among RCEP members has fluctuated over time, with a temporary spike during the COVID-19 pandemic, followed by a gradual normalization. The trade network does not exhibit small-world properties, indicating a relatively balanced trade relationship. Due to its resource advantages, China occupies a central role acting as a dominant producer and leading exporter. Vietnam’s export performance has been excellent in recent years, with the highest annual growth rate. Emerging markets, such as Myanmar, warrant closer attention. Economic size and population significantly positively affect trade value, while geographic distance and land adjacency have no significant impact. Trade activity is positively influenced by cultural and linguistic similarities, and countries with higher levels of economic freedom tend to engage in more trade. Tonifying TCM products with antioxidant and immune-boosting properties are more widely recognized in international markets.

**Conclusion:**

RCEP has established an excellent trade platform for the export of TCM. The factors influencing TCM trade are predominantly long-term and structural, rather than being driven by the occurrence of any single, isolated event. Member states should strengthen collaboration in standardization, technology coordination, and knowledge sharing to establish a mutually beneficial trade ecosystem for TCM.

## 1 Introduction

Traditional Chinese medicine (TCM) is not only an effective solution for primary health care, but also a great resource for drug innovation and discovery. TCM encompass a wide range of natural substances such as plants, animals, minerals, and their processed forms. TCM products include extracts, crude drug, herbal pieces, TCM preparations, health foods, and cosmetics (1). The efficacy of TCM has been observed in preventing and treating various ailments such as autoimmune disorders (2, 3), cardiovascular diseases (4) and cancers (5). The history of TCM trade dates back thousands of years. From the 2nd century BCE to the 14th century CE, China disseminated numerous medicinal herbs along the Silk Road to Central Asia, West Asia, Europe, and other regions. Tea, ginseng, ephedra, and goji berries became significant commodities along the Silk Road. Through this route, East Asian TCM culture began to interact and exchange with Western medical knowledge. From the 7th to the 13th century, during the Tang and Song dynasties, TCM spread to Japan, Korea, and Southeast Asia, where it had a profound impact on local medical practices. Between the 14th and 19th centuries, during the Ming and Qing dynasties, China expanded its trade of medicinal herbs such as ginseng, astragalus, and salvia through maritime routes, exporting them to Asia, the Americas, and Europe. Today, TCM has become a significant alternative or complementary treatment to Western medicine, with increasing international recognition and application. The recognition of Tu Youyou’s work and her Nobel Prize win in 2015 brought significant attention to the rich pharmacological effects of TCM on the global stage. Tu Youyou discovered artemisinin, derived from the plant *Artemisia annua*, which revolutionized malaria treatment (6). Artemisinin’s discovery highlighted the potential of TCM in modern medicine and fueled growing global interest in complementary and alternative medicine. While its primary use is in China, TCM also widely used in East Asia (e.g., Japan, Korea) and Southeast Asia (e.g., Vietnam, Thailand). Some European countries, such as Germany, France, and the United Kingdom, have incorporated aspects of Chinese medicine into their healthcare systems (7, 8). In Germany, for example, Chinese medicine practices such as acupuncture and herbal medicine are recognized and practiced alongside conventional medicine (9).

China boasts nearly 13,000 varieties of wild medicinal resources, with 163 new species discovered during the 4th National Survey of TCM resources (10). As a unique health and economic resource in China, TCM holds significant importance for the nation’s overall economic and social development and has received considerable attention from the government (11). In October 2016, the State Council issued the “Healthy China 2030” Plan, which comprehensively integrated TCM into the national health strategy. Subsequently, in February 2021, the State Council released a notice outlining several policy measures aimed at accelerating the trade of TCM. These measures include drafting the “14th Five-Year Plan” for TCM under the “Belt and Road” initiative and promoting the “Internet plus TCM trade” (12, 13). According to China Customs statistics, in 2023, the import and export value of pharmaceutical products in China reached 195.36 billion US dollars, representing 23% of the global pharmaceutical trade market. However, the TCM sector accounted for only 4% of China’s total pharmaceutical trade value, reflecting a year-on-year decline of 1.9%. Coincidentally, from January to May 2024, the trade value of TCM reached 3.461 billion US dollars, accounting for 4.3% of China’s total pharmaceutical trade value, representing a year-on-year decline of 3.65%. Although the Chinese government attaches great importance to and vigorously promotes the TCM trade, it has not achieved the desired effect in the actual trade performance.

The COVID-19 pandemic has introduced significant fluctuations in demand for medical supplies and medicine. Initial surges in demand were followed by subsequent declines, resulting in an overall impact on market stability (14, 15). The complex and volatile international political and economic landscape has further complicated matters. Developing a more open regional trade framework has become a key focus for many countries. The Regional Comprehensive Economic Partnership (RCEP) is a significant regional trade agreement led by Association of Southeast Asian Nations (ASEAN), characterized by a relatively high level of openness. First proposed at the East Asia Summit in November 2011, RCEP saw significant progress on November 15, 2020, when the ten ASEAN countries, along with China, Japan, New Zealand, South Korea, and Australia, signed the agreement. On January 1, 2022, RCEP came into effect, marking the development of the most free and open trade agreement to date (16). As a free trade agreement encompassing around 3.5 billion people and one-third of the global GDP, RCEP aims to establish a unified market across 15 countries (Indonesia, Malaysia, Philippines, Singapore, Thailand, Brunei, Vietnam, Laos, Myanmar, Cambodia, China, Japan, New Zealand, South Korea, and Australia) by reducing tariffs and non-tariff barriers. Some scholars posit that the signing of RCEP will foster a frequent regional trade. For example, since 2019, Vietnam’s TCM exports to China have shown consistent high growth. The export value has surged from millions to tens of millions of dollars, reaching $35.93 million in 2023. The main exported varieties include Areca nut, Sterculia lychnophora (Malva nut), Poria, and Cinnamon (17). Current trade data indicates that RCEP has contributed to promoting the TCM trade for some specific countries.

However, a comprehensive understanding of the scale, network characteristics, and determinants of TCM trade among RCEP members remains limited. Our research aims to answer the following questions: What is the scale and characteristics of TCM trade among RCEP member countries? Which countries play a pivotal role, and what factors enable them to influence trade? What are the main TCM export products with high recognition in international markets? The remainder of this paper is organized as follows: Section 2 elaborates the theoretical basis. Section 3 introduces the method and data sources. Section 4 focuses on empirical analysis and provides a reasonable explanation of the results. Section 5, 6 concludes discussion and conclusion, respectively.

## 2 Theoretical basis

The trade of TCM among RCEP member states are intricate and intertwined, forming a complex trade system. The characteristics and influencing factors of TCM trade networks among RCEP participating countries remain under-explored. Social Network Analysis (SNA) offers a robust framework for dissecting these complex trade relationships by constructing trade networks, extracting core subnets based on trade weights, and analyzing the structure and attributes of the interconnected nodes. Based on the literature review, Serrano and Boguná first applied SNA to international trade relations, and identified typical characteristics including scale-free distribution, small-world characteristics and high clustering coefficients (18, 19). Newman and Park discovered the topological characteristics of trade network, such as network density, clustering coefficients, and average path length (20). A further investigation carried by Fagiolo revealed a core-periphery structure that helps in determining the status of participating countries in trade networks (21).

The classical gravity mode have used to explore the factors affecting trade cooperation in previous studies. Hasson and Tinbergen were the first to apply the gravity model to international trade research (22). Subsequently, factor endowments (23), economic size (24), common borders (25) and regional trade agreements (26) have been incorporated into the gravity model. However, these studies only partially explain the influencing factors, and do not consider the interrelationships between these factors. Standard statistical procedures are inadequate for parameter estimation and statistical tests, due to the risk of calculating incorrect standard deviations. To address this issue, scholars employ randomized detection methods to test, and quadratic assignment procedure (QAP) is one such approach. QAP compares the similarity of each element in the two matrices, calculates the correlation coefficient between the matrices, and conducts non-parametric test on the coefficient (27, 28). QAP mitigates problems related to multi-collinearity and structural autocorrelation.

Spatial proximity, includes geographic distance and shared border, are considered the primary determinants affecting trade. The geographic proximity between economic entities significantly influences the formation of trade linkages by reducing transaction costs. Studies conducted by Anderson and Wincoop have demonstrated that closer physical distance often facilitates more efficient exchanges, thereby strengthening economic ties (29). Additionally, a shared land border is considered as a crucial factor in measuring trade cost, often serving as a proxy variable for geographical distance. The second factor is linguistic differences. Language can directly impact the way and cost of communication in international trade. Unimpeded communication facilitate a reduction in information acquisition costs and cognitive blind spots on both sides, leading to improved credit enhancement and increased international trade (30). The third factor is economy and population scale. Countries with larger economies and populations exhibit greater market demand and possess stronger production and export capacities, which in turn influence the flow and scale of trade (31). The fourth factor is Fixed Trade Costs (FTC). FTC refer to the non-variable expenses incurred by firms when entering and maintaining participation in international trade, regardless of the trade volume. These costs encompass expenses related to regulatory compliance, establishing distribution networks, and overcoming market entry barriers. Market access and regulatory systems vary across countries, influenced by distinct economic structures, non-tariff barriers, and levels of government intervention (32). Generally, countries with higher levels of economic freedom are associated with lower FTC.

Based on the above analysis, we propose the following hypotheses on the factors influencing TCM trade among RCEP members:

*Hypothesis 1 (H1)*. Countries that are geographically closer or with shared boundaries are more likely to trade with each other.

*Hypothesis 2 (H2)*. Countries with same language are more likely to trade with each other.

*Hypothesis 3 (H3)*. Countries with large economies and population scale have greater demand for TCM.

*Hypothesis 4 (H4)*. Countries with high economic freedom index are more likely to establish trade relations.

Over the past decade, researches combined SNA with QAP have accumulated substantial research experiences and cases, such as crude oil (33, 34), fossil energy (35, 36), electricity (37), etc. However, there is no relevant application of TCM trade. This paper aims to use SNA to establish TCM trade networks among RCEP members and study the network structures and node attributes. We also adopt QAP to explore the determinants of TCM trade networks among RCEP members.

## 3 Materials and methods

### 3.1 Research methodology

#### 3.1.1 Network construction

In this study, we utilize UCINET 6.504 software to construct a weighted network of TCM trade among RCEP members. Each node represents a country, the edge E_ij_ denotes the trade relation between country i and j. If there is no trade relationship between country i and j, then E_ij_ = 0, otherwise, E_ij_ = 1.The trade value from country i to j denoted as W_ij_ (38). Considering the asymmetries in the trade data, we adopt the maximum value as the weight of the edge between country i and j.

#### 3.1.2 Statistic indicators

To characterize the structural properties of the network, this paper sets up two levels of indicators. The first tier outlines the overall structural features. Network density (*D*) quantifies the extent to which nodes are interconnected, with higher density signifying a greater intensity of commercial activity. Average path length (*L*) represents the mean number of intermediary nodes traversed within the network when moving from one node to another. It is a critical metric for evaluating the efficiency of information or resource flow within a network, reflecting the overall connectivity and potential bottlenecks in the system. The clustering coefficient (*C*) reflects the extent to which a node’s connections are tightly knit with its neighboring nodes. If the network has a shorter *L* and a larger *C*, the network exhibits small world property, indicating efficient connectivity with localized clusters (39). The second tier of indicators describes the structural attributes of individual nodes, focusing on node degree and betweenness centrality. Node degree (*K*) refers to the number of direct connections a specific node has within the network. In a directed network, this can be further categorized into out-degree and in-degree. The node degrees for 2013, 2018, and 2023 were ranked from smallest to largest in this study. Betweenness centrality (*BC*) measures the likelihood that the shortest paths between nodes pass through a particular node, reflecting its control capability within the network (Table 1).

**Table 1:** Indicators of the network structure.

Indicators	Equation	Description
Density (*D*)	$D=\frac{M}{N\left(N-1\right)}$	*M* is the number of edges in the network. *N* indicates the total number of network nodes.
Average Path Length (*L*)	$L=\frac{1}{N\left(N-1\right)}\underset{i\neq j}{\sum }d_{ij}$	*d_ij_* reflects the minimum number of edges in all paths from *i* to *j*.
Clustering coefficient (C)	$C=\frac{1}{N}\overset{N}{\underset{i=1}{\sum }}C_{i}$ $C_{i}=2X_{i}/K_{i}\left(K_{i}-1\right)$	*X_i_* is the actual number of connections among *i*’s neighbors.
Node-Degree (*K*)	$K_{i}\left(t\right)=K_{i}^{\mathrm{out}}+K_{i}^{\mathrm{in}}$ $K_{i}^{\mathrm{out}}\left(t\right)=\overset{N\left(t\right)}{\underset{j=1}{\sum }}a_{ij}\left(t\right)$ $K_{i}^{\mathrm{in}}\left(t\right)=\overset{N\left(t\right)}{\underset{j=1}{\sum }}a_{ji}\left(t\right)$	*a_ij_* denotes the number of nodes from *i* to *j*, *a_ji_* denotes the number of nodes from *j* to *i*.
Betweenness Centrality(*BC*)	$BC_{i}=\frac{2\underset{jk}{\sum }g_{jk}\left(i\right)/g_{jk}}{n^{2}-3n+2}$	*g_jk_* is the number of shortcuts between *j* and *k*, and *g_jk_*(*i*) is the number of shortest paths between *j* and *k* through *i*.

#### 3.1.3 Core-peripheral analysis

Core-peripheral analysis is used to study the contribution and status of participating countries in the networks. This approach facilitates to identify closely connected centers as well as scattered peripheries. Core regions typically consist of member countries with high trade activity and strong interconnections, whereas peripheral regions are characterized by fewer and more scattered trade linkages. The algorithm for core-peripheral analysis was first proposed by Borgatti and Everett and can be categorized into discrete and continuous model (40, 41). In this study, we adopt a continuous core-peripheral model by calculating the cores of each node. The specific calculation formula is as follows:

$$\rho =\underset{ij}{\sum }a_{ij}\delta_{ij}\left(\delta_{ij}=c_{i}\times c_{j}\right)\text{.}$$

In calculation formula, *C_i_* and *C_j_* represent the cores of nodes i and j, respectively. *δ_ij_* represents the element of the pattern matrix *δ* corresponding to the ideal core-edge model, while *a_ij_* represents the element of the actual adjacency weight relation matrix. The correlation index *ρ* measures the correlation between the pattern matrix and the actual adjacency matrix. When *ρ* reaches the maximum value, *δ* represents the edge-core structure matrix that closely approximates the actual situation and corresponds to the nearest quasi-ideal model (42).

#### 3.1.4 QAP analysis and factor selection

QAP analysis is a randomized detection method that consists of correlation analysis and regression analysis. The correlation analysis examines the relationship between each influencing factor and the trade network, while the regression analysis investigates the statistical significance and magnitude of the influencing factors (43). The QAP algorithm proceeds in three steps. Firstly, it calculates the Pearson correlation coefficient between corresponding cells of the two data matrices. Secondly, it randomly permutes rows and columns of one matrix and recalculates the correlation and other measures. Lastly, step 2 is repeated thousands of times to determine the proportion of times that the randomly generated measure is equal to or greater than the observed measure calculated in step 1.

Based on the QAP model, this paper selects four primary variables to analyze the factors influencing the export flow of TCM products among RCEP members. The model constructed in this study is as follows:

$$T=f\;\left(\begin{array}{l} \mathrm{Diff}\_\mathrm{distance,Binary}\_\mathrm{border,Binary}\_\mathrm{language,}\;\\ \mathrm{Diff}\_\mathrm{GDP,}\;\mathrm{Diff}\_\mathrm{population,Diff}\_\mathrm{economic}\;\\ \mathrm{freedom index} \end{array}\right)\text{.}$$

where the dependent variable T represents the matrix of TCM trade network, Diff_distance, Diff_GDP, Diff_population and Diff_economic freedom index are matrices that represent the absolute differences in the corresponding indexes. These four variables are standardized by the columns of the matrix. Binary_border and Binary_language are binary matrices. If two countries are the same, the value takes 1, otherwise it takes 0.

### 3.2 Data resource

The TCM trade data of RCEP members were extracted from the UN-Comtrade database spanning from 2013 to 2023. The specific trade data is identified by the HS code. HS121190, which representing plants and parts of plants, used primarily in pharmacy, fresh or dried, whether or not cut, crushed or powdered. HS1302.19, which typically referring to plant extracts not specified for a particular use, including but not limited to natural gums, resins, and other plant extracts. HS 3301.90, which pertains to extracts and derivatives of aromatic plants, commonly used in perfumes, cosmetics, and food additives. Re-export and re-import quantities were not considered, because of their tiny proportion in the overall trade. Table 2 presents the variables used in QAP analysis. Data on GDP and total population were acquired from the World Bank database. Geographical distance, land boundary, and language were obtained from the Cep II database. The economic freedom index, published by the American Heritage Foundation, provides a comprehensive assessment of FTC by evaluating trade policies, government intervention, monetary policy, and the financial sector in 155 countries.

**Table 2:** Variables, description, and data source of QAP model.

Variable	Description	Source
Geographical Distance	Absolute value of the difference in distance between the two capitals	http://www.cepii.fr
Land adjacency	Whether there is a common geographical boundary contiguity	http://www.cepii.fr
GDP	Absolute value of GDP difference	World Bank Open Data | Data
Population	Absolute value of population difference	World Bank Open Data | Data
Language	Whether there is a common language	CEPII – Accueil
Fixed Trade Costs	Absolute value of economic freedom index difference	https://www.heritage.org/index/pages/country-pages/

## 4 Results

### 4.1 An overview of TCM trade

**Figure 1:**
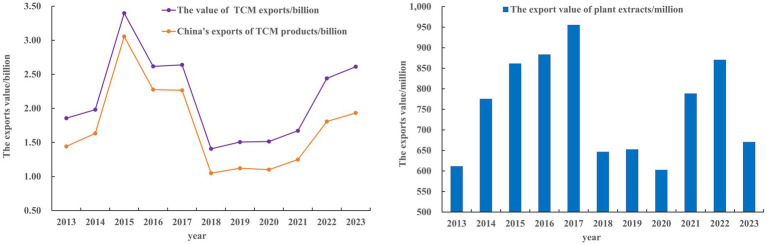
Changes in the value of TCM exports among RCEP members, variation in China’s exports of TCM products, and fluctuations in the export value of plant extract.

The TCM trade value among RCEP members has undergone three notable phases. From 2013 to 2015, TCM exports experienced a rapid growth period, surging from $1.856 billion to a peak of $3.397 billion. This was followed by an adjustment phase from 2016 to 2020, during which the export value decreased to around $1.5 billion. Despite the positive global impact of Tu youyou’s discovery of artemisinin, which earned her Nobel Prize in 2015, TCM trade did not show significant growth between 2016 and 2018. This could be attributed to the complexity and diversity of TCM, which poses substantial challenges to its standardization and normalization during the process of internationalization. Although Tu Youyou’s discovery highlighted the substantial potential of TCM in modern medical applications, its use remains primarily limited to specific fields, such as malaria treatment. Overall, TCM still lacks sufficient clinical trials and contemporary scientific evidence to substantiate its efficacy, leading to its limited acceptance in global markets, particularly in Europe and the United States. Starting in 2020, the COVID-19 pandemic led to a significant increase in demand for TCM, driving export values up to $2.612 billion. The export value of plant extracts, the main subcategory of TCM, has consistently increased, culminating in a peak of $870 million in 2022. However, in 2023, the first year of normalized post-pandemic conditions, the global market has exhibited a general decline in demand coupled with a trend towards inventory reduction. As a result, the plant extract industry has experienced the dissipation of the “pandemic dividend,” leading to a substantial year-on-year decrease in export volumes (Figure 1).

**Figure 2:**
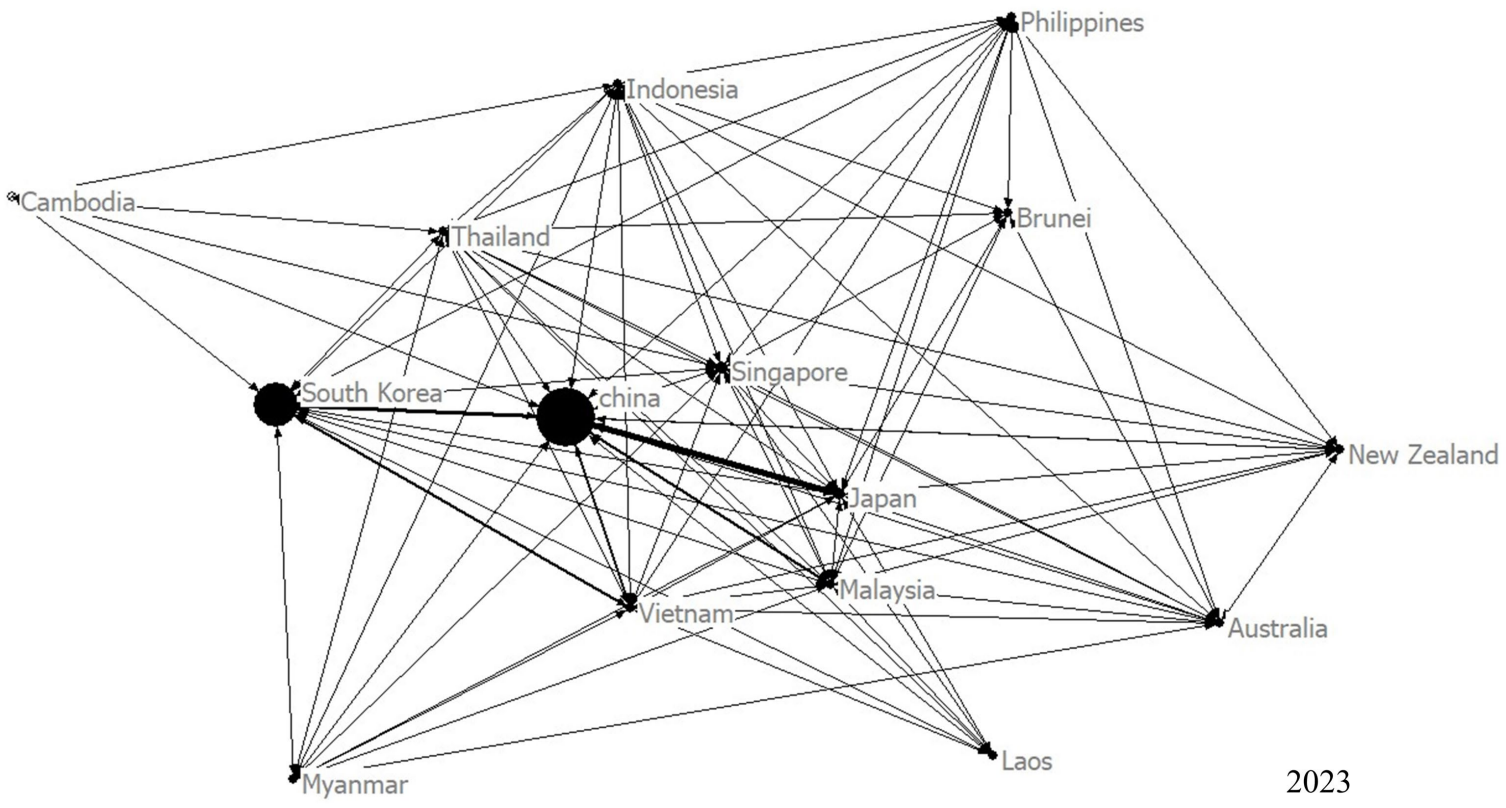
The network of TCM trade among RCEP members in 2023.

**Figure 3:**
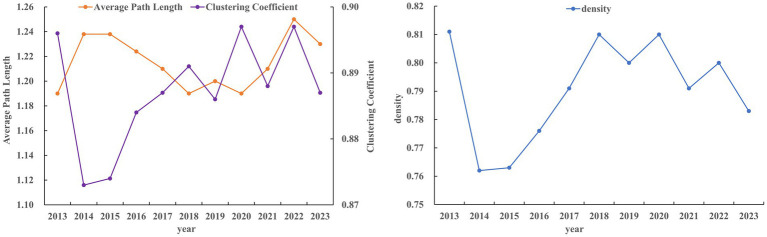
Network density (D), average path length (APL) and Clustering coefficient (C) from 2013 to 2023.

To gain a comprehensive understanding of the trade relationships among RCEP members, we constructed a visualized TCM trade network, and analyzed several key indicators. Figure 2 shows the network of TCM trade among RCEP member countries in 2023. The network density remained relatively stable, with a maximum value of 0.811 in 2022 and a minimum of 0.762 in 2014, indicating close trade relationships among RCEP members. Additionally, the *L* value ranged from 1.19 to 1.25, while the *C* value varied between 0.873 and 0.897 (Figure 3). Notably, the maximum *L* value was only marginally higher than the minimum *C* value, suggesting that the TCM trade network does not exhibit small-world characteristics, thereby reflecting a well-balanced trade relationship among the RCEP member states.

### 4.2 Nodes attribution

In this study, the node degree was ranked from small to large, and the top 5 nodes in 2013, 2018 and 2023 were identified and shown in Table 3. Although the node degree ranking varies in different times, some key countries, for example, China, South Korea, Japan, Singapore and Thailand remained at the forefront of ranking. The results indicate their relatively large market demand, great export advantage and strong market control ability. Among the RCEP members, China plays a central role in the TCM trade network, given its position as both a leading producer and exporter of TCM products. China’s extensive production capabilities, coupled with its established trade relations, enable it to influence trade patterns significantly. From 2013 to 2023, the export value of TCM, encompassing both medicinal materials and plant extracts, increased from $1.4424 billion to $1.933 billion, reflecting a compound annual growth rate (CAGR) of 3.4% (Figure 1). Notably, during the COVID-19 pandemic (2020–2023), TCM exports exhibited a marked acceleration, with an average annual growth rate of 25.2%. The principal export destinations are always in Japan, South Korea, and Australia. In 2023, South Korea for 8.18% of the total export value, followed by Japan (5.18%), Vietnam (3.67%), Thailand (3.38%). On the import front, Indonesia and Vietnam emerged as significant contributors. Imports from Indonesia reached a peak value of $1.43 billion. In comparison, imports from Vietnam amounted to $0.36 billion, demonstrating the highest year-on-year growth rate of 78% (see Table 4).

**Table 3:** The top 5 nodes in 2013, 2018, and 2023.

Year	In-degree		Out-degree		Betweenness	
	Country	Degree	Country	Degree	Country	Degree
2013	China	13	Indonesia	14	Malaysia	3.701
	Japan	12	Malaysia	14	Thailand	3.701
	Singapore	12	Thailand	14	Indonesia	3.701
	South Korea	11	Singapore	13	Singapore	3.701
	Thailand	11	Australia	12	Australia	1.783
2018	China	13	Thailand	14	China	3.375
	Singapore	13	South Korea	14	South Korea	3.375
	Thailand	12	Singapore	14	Thailand	3.375
	Malaysia	12	China	13	Singapore	3.375
	Japan	12	Malaysia	13	Japan	2.311
2023	Malaysia	12	Thailand	14	Singapore	4.294
	China	11	Singapore	14	Thailand	4.294
	Japan	11	South Korea	13	Malaysia	2.960
	Singapore	11	China	13	China	2.842
	Thailand	11	Malaysia	13	South Korea	2.842

**Table 4:** Distribution of China’s top three export markets.

Year	Export markets /proportion				
	No 1	No 2	No 3	No 4	No 5
2013	Australia (21.44%)	South Korea (10.4%)	Japan (9.56%)	Vietnam (3.71%)	Malaysia (2.87%)
2014	Australia (14.00%)	South Korea (11.2%)	Japan (8.56%)	Vietnam (3.44%)	Malaysia (3.27%)
2015	South Korea (13.59%)	Australia (11.97%)	Japan (7.79%)	Singapore (5.2%)	Malaysia (3.8%)
2016	South Korea (10.68%)	Australia (8.98%)	Japan (8.12%)	Thailand (4.6%)	Malaysia (2.8%)
2017	Australia (9.98%)	South Korea (8.17%)	Japan (7.84%)	Malaysia (5.8%)	Vietnam (3.47%)
2018	South Korea (9.68%)	Australia (8.38%)	Japan (7.12%)	Singapore (5.45%)	Malaysia (3.08%)
2019	South Korea (10.68%)	Japan (7.02%)	Australia (6.38%)	Vietnam (3.37%)	Malaysia (2.87%)
2020	South Korea (9.68%)	Japan (6.02%)	Australia (5.83%)	Thailand (4.88%)	Vietnam (3.07%)
2021	South Korea (9.08%)	Australia (6.38%)	Japan (5.12%)	Vietnam (3.27%)	Indonesia (2.9%)
2022	South Korea (7.08%)	Australia (6.08%)	Japan (5.13%)	Thailand (4.38%)	Vietnam (2.67%)
2023	South Korea (8.18%)	Japan (5.18%)	Vietnam (3.67%)	Thailand (3.38%)	Indonesia (2.87%)

### 4.3 Core-periphery structure analysis

The RCEP member countries were divided into three categories through the core value (Table 5). The countries with core value greater than 0.3 were considered as core, those between 0.2 and 0.3 were considered as semi-periphery, and those less than 0.2 were considered as periphery. By calculating the core values of RCEP member countries from 2013 to 2023, we found that nations with core values exceeding 0.3 are predominantly clustered in Singapore, Thailand, South Korea, and China. Conversely, those with core values below 0.2 are primarily found in Laos, Myanmar, Brunei, and Cambodia. Additionally, the two Oceania members, Australia and New Zealand, are positioned within the semi-core region, with New Zealand exhibiting marginally lower levels of activity. These findings indicate that, while it is crucial to maintain stable trade relations with core nations, there is significant strategic potential in expanding market engagement with countries such as Laos, Myanmar, Brunei, Cambodia, and New Zealand.

**Table 5:** The RCEP member countries in core value.

2013		2018		2023	
Country	Corene	Country	Corene	Country	Corene
China	0.299	South Korea	0.301	Singapore	0.304
Singapore	0.299	China	0.301	Thailand	0.304
Thailand	0.299	Thailand	0.301	Japan	0.293
Vietnam	0.286	Singapore	0.301	Myanmar	0.293
Australia	0.286	Malaysia	0.287	Indonesia	0.288
Malaysia	0.286	Japan	0.283	South Korea	0.288
South Korea	0.286	Vietnam	0.283	China	0.288
Japan	0.274	Australia	0.277	Australia	0.277
Indonesia	0.269	Philippines	0.272	Vietnam	0.277
Philippines	0.257	Indonesia	0.253	Philippines	0.257
Myanmar	0.248	New Zealand	0.237	New Zealand	0.242
New Zealand	0.235	Myanmar	0.213	Myanmar	0.218
Cambodia	0.188	Brunei	0.185	Laos	0.167
Brunei	0.139	Cambodia	0.166	Brunei	0.165
Laos	0.137	Laos	0.141	Cambodia	0.119

### 4.4 Analysis of influencing factors

#### 4.4.1 QAP correlation analysis

In this paper, 5,000 random permutations were conducted and TCM trade matrix among RCEP members and its impact factors in 2013, 2018 and 2023 were selected for correlation analysis.

Analysis reveals that land borders, GDP, population, and language significantly impacted TCM trade in 2013, 2018, and 2023, with these variables showing statistical significance at the 1% level (Table 6). Geographic distance were the factors negatively correlated with the trade network, indicating that greater distances correspond to lower trade volumes. The economic freedom index exhibited a positive correlation, indicating FTC were also negatively correlated with trade.

**Table 6:** QAP correlation analysis results.

Variables	2013	2018	2023
Diff_Geographic distance	−0.101	−0.056	−0.081
Diff_Land borders	0.235^***^	0.196^**^	0.079
Diff_GDP	0.280^***^	0.311^***^	0.207^***^
Diff_Population	0.188^*^	0.304^**^	0.238^**^
Diff_Language	0.301^***^	0.228^**^	0.196^**^
Diff_economic freedom index	0.126	0.191	0.155

^*^p < 0.1, ^**^p < 0.05 and ^***^p < 0.01.

#### 4.4.2 QAP regression analysis

Considering correlation analysis only provided preliminary results, QAP regression analysis was then applied to further investigate the statistical significance of the six explanatory variables. The regression results in 2013, 2018 and 2023 are shown in Table 7.

**Table 7:** Results of QAP regression.

Variables	2013	2018	2023
Diff_Geographic distance	−0.046	−0.102	−0.120
Diff_Land borders	0.096	0.036	0.079
Diff_GDP	0.280^***^	0.311^***^	0.336^***^
Diff_Population	0.035^**^	0.136^**^	0.046^**^
Diff_Language	0.235^***^	0.228^***^	0.116^*^
Diff_economic freedom index	0.037	0.123	0.101

^*^p < 0.1, ^**^p < 0.05 and ^***^p < 0.01.

Firstly, geographic distance and land adjacency did not show significant effects on TCM trade. Since most RCEP members are located in Asia, with major regions such as some ASEAN countries sharing borders, the impact of spatial distance on trade is minimal. In addition, transportation, such as marine and air freight also reduce the restrictions of space distance on trade. Secondly, the coefficient of economy and population scale were positive and statistically significant. TCM products are both resource-intensive and labor-intensive. A developed economy often reflects advanced technical processing capabilities and consumption power, while a large population not only signifies greater demand but also provides a substantial labor force engaged in TCM processing. These factors collectively enhance the flow of trade in TCM products. Thirdly, language were the positive elements and statistically significance. This finding implied that TCM trade benefited from a similar culture background, with the countries with same language are more likely to trade with each other. Finally, economic freedom index show positive effect on trade, which means countries with higher levels of economic freedom have higher trade activity.

In addition to considering the above-mentioned factors, we also explored whether there is a connection between medicinal resources and TCM trade. The RCEP member countries are rich in medicinal plant resources. We obtained a list of plant species and their geographic distribution records for RCEP member countries from Plants of the World Online (POWO, https://powo.science.kew.org). The scientific names of the medicinal plants from the RCEP member countries were cross-referenced with the Medicinal Plant Names Services system (MPNS, https://www.kew.org/science/our-science/science-services/medicinal-plant-names-services), which allowed us to estimate the approximate number of medicinal plant species in these countries. Table 8 demonstrates that China possesses a relatively abundant supply of medicinal plant resources, which confers a natural advantage in the provision of raw materials for TCM trade. These resource advantages may be another factor contributing to China’s leadership in the TCM trade within the RCEP member countries.

**Table 8:** The approximate number of plant species and medicinal plants, and the representative herbs in RCEP countries.

Country	Plant species	Medicinal plant species	Representative herbs
China	34,180	11,146	*Panax ginseng*, *Lycium barbarum*, *Astragalus membranaceus*, *Angelica sinensis*, etc.
Singapore	9,550	365	*Perilla frutescens*, *Glycyrrhiza glabra*, *Coptis chinensis*, etc.
Thailand	11,714	1712	*Zingiber officinale*, *Cymbopogon citratus*, *Momordica charantia*, etc.
Vietnam	12,715	3,320	*Momordica charantia*, Mentha spp., Gentiana scabra, etc.
Australia	24,165	550	Melaleuca alternifolia, Eucalyptus spp., *Macadamia integrifolia*, etc.
Malaysia	2,640	1,156	*Zingiber officinale*, Mentha spp., *Momordica charantia*, etc.
South Korea	3,920	680	*Panax ginseng*, *Astragalus membranaceus*, *Angelica sinensis*, etc.
Japan	6,432	883	Glycyrrhiza uralensis, *Lycium chinense*, *Perilla frutescens*, etc.
Indonesia	1,550	1,134	*Curcuma longa*, *Zingiber officinale*, *Momordica charantia*, etc.
Philippines	4,940	867	*Glycyrrhiza glabra*, *Momordica charantia*, *Piper nigrum*, etc.
Myanmar	10,180	3,635	*Achyranthes bidentata*, *Momordica charantia*, *Zingiber officinale*, etc.
New Zealand	3,747	210	Rongoa Māori, Kawakawa, etc.
Cambodia	4,246	716	*Momordica charantia*, *Zingiber officinale*, *Glycyrrhiza glabra*, etc.
Brunei	2,332	220	*Cinnamomum verum*, *Gentiana scabra*, *Gentiana scabra*, etc.
Laos	5,486	836	*Coptis chinensis*, *Glycyrrhiza glabra*, *Ocimum basilicum*, etc.

### 4.5 Major export products

Taking China as an example, we summarize the main TCM products exported in recent years. These exported TCM products include plant extracts, medicinal herbs, Chinese patent medicines, including compound formulations and Chinese Medicine Granules and Oral Liquids. As shown in Table 9, among the RCEP member countries, TCM products containing ginseng, goji berries, astragalus, and *Salvia miltiorrhiza*, are widely accepted in foreign markets due to their antioxidant properties and immune-enhancing effects.

**Table 9:** The main exported TCM products.

Exported TCM products	Representative drugs	Pharmacological actions
1. Plant extracts 2. Medicinal herbs	Ginseng	Ginseng contains ginsenosides, total ginsenosides, and ginseng polysaccharides, and is widely used in health products for boosting immunity, anti-fatigue, and anti-aging.
	Goji Berry	Goji berry is rich in natural antioxidants, particularly goji polysaccharides and carotenoids, and is commonly used in dietary supplements, functional foods, and anti-aging products.
	Reishi Mushroom	Reishi mushroom is widely used in immune regulation, anti-tumor, and anti-fatigue fields. Its main active ingredients, such as reishi polysaccharides and triterpenoids, are in high demand in the international market.
	Astragalus	Astragalus primarily consists of astragalus polysaccharides, which have functions such as boosting immunity, anti-fatigue, and antioxidant properties. It is commonly used in the health supplement and pharmaceutical industries.
	*Salvia miltiorrhiza*	*Salvia miltiorrhiza* extract (e.g., tanshinone) is often used in products that improve blood circulation, provide antioxidant and anti-inflammatory effects, and has potential applications in the treatment of cardiovascular diseases.
3. Compound formulations	Compound Danshen Dripping Pills	Used for treating cardiovascular and cerebrovascular diseases, especially coronary heart disease and angina.
	Anshen Buxin Pills	Used for treating symptoms such as insomnia, anxiety, and palpitations.
	Baoji Pills	Used for digestive system issues, such as diarrhea and stomach pain.
4. Chinese Medicine Granules and Oral Liquids	Compound Honeysuckle Granules	Used for antibacterial and antiviral purposes, commonly used to treat colds and upper respiratory infections.
	Dandelion Oral Liquid	Often used for clearing heat and detoxifying, treating skin inflammation or liver issues.

## 5 Conclusion and discussion

### 5.1 Conclusion

The TCM trade among RCEP member countries exhibits distinct characteristics compared to other commodity trade. Firstly, TCM trade did not experience a substantial surge following Tu Youyou’s Nobel Prize award. However, the COVID-19 pandemic induced a temporary spike in exports, which later returned to pre-pandemic levels. This trend indicates that the factors influencing TCM trade are primarily long-term and structural in nature, rather than being driven by the occurrence of any single, isolated event. Secondly, the trade network of TCM does not exhibit small-world characteristics, indicating a relatively balanced trade relationship among member states. Thanks to its resource advantages, China occupies a pivotal position in the TCM trade network, being both a dominant producer and a leading exporter of TCM products. Vietnam’s export performance in recent years has been exceptional, with the highest year-on-year growth rate among member nations. Countries such as Laos, Myanmar, Cambodia, and New Zealand possess substantial untapped market potential. Thirdly, economic size and population scale exert a significant positive influence on trade value, whereas geographic distance and land adjacency appear to have no statistically significant effects. Trade activity is enhanced by cultural and linguistic similarities, and countries with higher levels of economic freedom tend to exhibit greater trade activity. Tonifying TCM products with antioxidant and immune-boosting properties are more widely recognized in international markets.

### 5.2 Discussion

#### 5.2.1 Commerce and conservation

The interplay between resource sustainability and trade requires careful consideration. The prudent management of resources is essential for the long-term sustainability of the economy, while patterns of economic development influence the dynamics of resource utilization (44). Trade should not advance at the expense of species extinction. It is recommended to enhance the protection of TCM resources by adopting environmentally friendly cultivation and harvesting practices. This approach will ensure a sustainable supply of raw materials and minimize negative environmental impacts.

#### 5.2.2 Dominant markets and emerging markets

While TCM dominates the Asian market, its potential in emerging markets such as Europe, the Americas, and Africa is growing with the rising global awareness of health and the popularity of natural therapies. RCEP offers opportunities for TCM to enter emerging markets, such as Southeast Asia and Oceania. In these markets, conducting cross-border marketing campaigns and strengthening collaborations with local distributors can help TCM companies establish stable sales channels. Additionally, trade exhibitions and seminars among RCEP member countries can serve as platforms to showcase the uniqueness and efficacy of TCM, thereby enhancing global consumer recognition and acceptance of these products.

#### 5.2.3 Regional cooperation and policy coordination

RCEP encompasses 15 countries across East Asia, Southeast Asia, and Oceania, offering a vast market. By leveraging the trade agreements and tariff concessions among RCEP member states, TCM enterprises can reduce tariff barriers, streamline approval processes, and enhance market access for their products. It is also important to note that due to varying perceptions and quality standards of TCM across different countries, market entry barriers for TCM remain relatively high. Promoting coordination among RCEP member countries in TCM production, testing, and regulatory standards to establish regionally or internationally recognized TCM standards (45). Governments should strengthen policy coordination, simplify the import and export procedures for TCM products, and facilitate smoother trade flows among member countries.

## Data Availability

The datasets presented in this study can be found in online repositories. The names of the repository/repositories and accession number(s) can be found in the article/supplementary material.
